# Evaluation of cyclophosphamide for steroid-refractory hepatic acute graft-vs-host disease after allogeneic hematopoietic stem cell transplantation

**DOI:** 10.3389/fimmu.2026.1678723

**Published:** 2026-03-02

**Authors:** Ni Lu, Yawei Zheng, Wenwen Guo, Xinhui Zheng, Dan Feng, Yigeng Cao, Rongli Zhang, Weihua Zhai, Donglin Yang, Jialin Wei, Yi He, Aiming Pang, Sizhou Feng, Mingzhe Han, Erlie Jiang

**Affiliations:** 1 State Key Laboratory of Experimental Hematology, National Clinical Research Center for Blood Diseases, Institute of Hematology & Blood Diseases Hospital, Chinese Academy of Medical Sciences & Peking Union Medical College, Tianjin, China; 2 Haihe Laboratory of Cell Ecosystem, Institute of Hematology & Blood Diseases Hospital, Chinese Academy of Medical Sciences & Peking Union Medical College, Tianjin, China; 3 Tianjin Institutes of Health Science, Tianjin, China

**Keywords:** allogeneic hematopoietic stem cell transplantation, cyclophosphamide, liver, salvage treatment, steroid-refractory acute graft-versus-host disease

## Abstract

**Introduction:**

Steroid-refractory (SR) hepatic acute graft-versus-host disease (aGVHD) remains a life-threatening complication following allogeneic hematopoietic stem cell transplantation, characterized by limited responsiveness to both first- and second-line therapies and an overall poor prognosis. This study aimed to evaluate the efficacy and safety of cyclophosphamide (CTX) as a salvage treatment for SR- hepatic aGVHD.

**Methods:**

A total of 50 patients with SR-hepatic aGVHD who underwent CTX treatment were retrospectively included in the analysis. Seventeen patients (34.0%) received CTX as second-line therapy, whereas the majority (n=33, 66.0%) were administered CTX as salvage therapy following failure of prior second-line interventions.

**Results:**

The overall response rate (ORR) at day 28 was 70.0%, with a durable ORR of 66.0% at day 56. Patients with the hepatitic variant of aGVHD showed a superior response compared to those with the classic variant (complete response: 6 of 8 [75.0%] vs. 14 of 42 [33.3%], P = 0.042). The probabilities of overall survival (OS) and nonrelapse mortality (NRM) at 3 years after CTX treatment were 36.9% (95% CI, 24.8%–54.9%) and 56.5% (95% CI, 41.4%–71.6%). Using propensity score matching (PSM), we compared 35 patients receiving CTX with 35 BAT (best available treatment) controls during the same study period. CTX initiation occurred later than BAT (median line: 3 vs 2, P < 0.001). Response rates and survival outcomes were comparable between two groups and CTX demonstrated consistent efficacy even when used as later-line therapy. Additionally, CTX did not significantly increase the risk of adverse events compared to the BAT group up to day 28. The most common adverse events in both groups were neutropenia (71.4% in the CTX group vs. 62.9% in the BAT group, P = 0.445), anemia (68.6% vs. 60.0%, P = 0.454), and cytomegalovirus infection (51.4% vs. 45.7%, P = 0.632).

**Discussion:**

These findings suggest that CTX is a promising and well-tolerated treatment option for patients with SR-hepatic aGVHD.

## Introduction

Allogeneic hematopoietic stem cell transplantation (allo-HSCT) remains the most effective intervention for achieving sustained remission in patients with high-risk myeloid malignancies and severe bone marrow failure (1). However, its clinical application is limited by the occurrence of graft-versus-host disease (GVHD), with acute GVHD (aGVHD) being the second leading cause of mortality in allo-HSCT patients, after relapse of the primary malignancy (2, 3). The liver is one of the most commonly affected organs in aGVHD and is an independent risk factor for increased non-relapse mortality (NRM) and poor overall survival (OS) (4). Hepatic aGVHD is characterized by progressive cholestasis, primarily manifested as elevated serum total bilirubin (≥2 mg/dL) and alkaline phosphatase. The standard first-line treatment for aGVHD involves systemic high-dose corticosteroids, with or without the addition of a calcineurin inhibitor (5). However, only 30–50% of patients with hepatic aGVHD experience resolution of liver abnormalities after initial therapy, and more than half of these patients subsequently develop chronic GVHD (cGVHD) (6, 7). The optimal salvage strategy for steroid-refractory (SR) aGVHD remains a subject of ongoing debate (8). In recent years, the therapeutic landscape has evolved with the approval of ruxolitinib, a JAK1/2 inhibitor, which demonstrated superior overall response rates compared to best available therapies in the landmark REACH2 trial (9). Other emerging modalities, such as mesenchymal stem cells (MSCs), alpha-1 antitrypsin, basiliximab, and Xenopax have also shown promise in modulating alloreactive immune responses (10–13). However, the liver remains the most recalcitrant organ to these interventions. Furthermore, the substantial financial burden and restricted global accessibility of these novel biologics pose significant barriers to their widespread clinical application. Given these multifaceted challenges, there is a critical need for cost-effective, biologically sound, and widely accessible interventions. Cyclophosphamide (CTX), recognized for its potent ability to modulate T-cell alloreactivity, emerges as a promising candidate to address this unmet clinical need.

Cyclophosphamide is a potent immunosuppressive antineoplastic agent and a cornerstone in the treatment of various hematologic malignancies (14, 15). It is routinely incorporated into pretransplant conditioning regimens to prevent graft rejection by suppressing the recipient’s immune response. High-dose post-transplant cyclophosphamide (PTCy) is widely used to prevent GVHD. Recent mechanistic studies have demonstrated that PTCy prevents GVHD by limiting functional alloreactivity in donor T cells and promoting a more rapid recovery of regulatory T cells, thereby inducing immune tolerance and reducing inflammation (16). Despite its extensive use in GVHD prevention, there is limited understanding of the application of CTX in treating aGVHD. In 2005, J. Mayer reported that pulse CTX, administered at a median dose of 1 g/m², was highly effective in treating SR-aGVHD. Furthermore, J.Mayer confirmed that CTX was effective in managing SR-hepatic aGVHD, achieving an overall response rate (ORR) of 80.9%, with a favorable toxicity profile (17). In a subsequent study, CTX was also shown to be effective in treating SR-hepatic aGVHD in a pediatric and a female patient (18, 19). Given the limited clinical data on the use of CTX in SR-hepatic aGVHD, the present study aims to systematically evaluate the clinical response to CTX and its safety profile in patients with SR-hepatic aGVHD following allo-HSCT.

## Methods

### Study design and patient population

**Figure 1:**
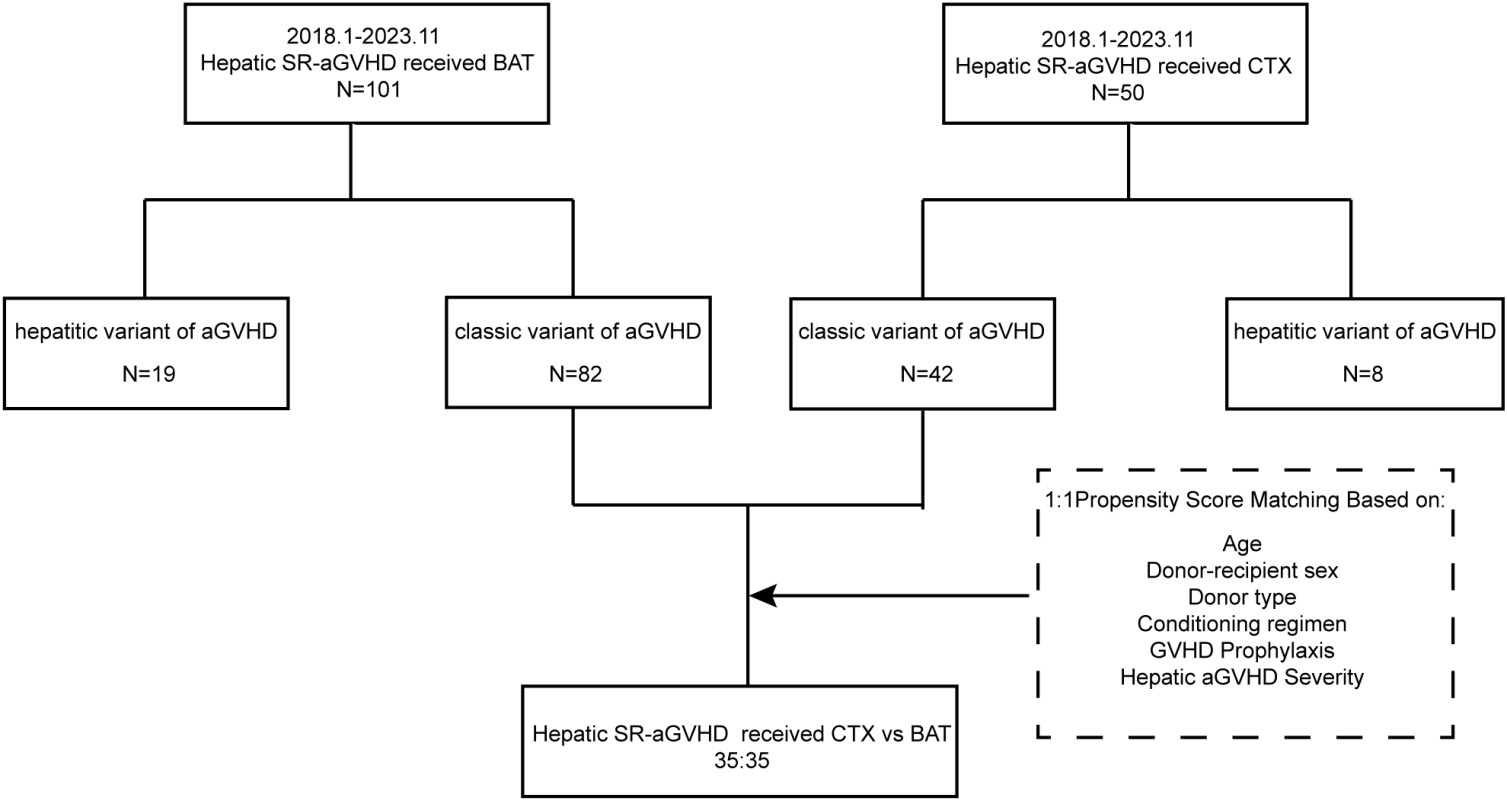
Flow diagram of the selection of study subjects. SR-aGVHD, steroid-refractory acute graft-versus-host disease; CTX, cyclophosphamide; BAT, best available therapy.

This retrospective, single-center study was conducted at the Institute of Hematology and Blood Diseases Hospital, Chinese Academy of Medical Sciences and Peking Union Medical College (IHCAMS), as part of the National Longitudinal Cohort of Hematological Diseases (NICHE; IIT2021011-EC-1). A total of 50 patients with SR-hepatic aGVHD who received CTX as second-line or later therapy between January 2018 and November 2023 were included (**Figure 1**).

Eligible patients included those with SR-hepatic aGVHD following allo-HSCT, regardless of underlying disease or donor type. Hepatic aGVHD was biochemically defined based on the MAGIC criteria: the classic variant required total bilirubin ≥ 2 mg/dL, while the hepatitic variant was characterized by ALT/AST> 5 ULN. SR status was defined as disease progression within 3 days or lack of improvement within 7 days of high-dose corticosteroids (≥2 mg/kg/day methylprednisolone). Patients were excluded if they had disease relapse, uncontrolled infections, or severe comorbidities at CTX initiation. Furthermore, given that liver biopsy was often clinically precluded by coagulopathy or thrombocytopenia, we employed a rigorous differential diagnostic algorithm to exclude confounding hepatopathies. Specifically, Sinusoidal Obstruction Syndrome (SOS) was ruled out via EBMT criteria (20) (monitoring weight, ascites, and hepatomegaly), and Transplant-Associated Thrombotic Microangiopathy (TMA) was excluded by the absence of schistocytes, stable LDH, and normal haptoglobin. Viral hepatitis (HBV, HCV, CMV, HSV, VZV) was systematically ruled out through PCR-based surveillance, while drug-induced liver injury (DILI) was adjudicated by assessing the temporal relationship with hepatotoxic agents.

A control cohort consisted of 82 patients with SR-hepatic aGVHD who received best available therapy (BAT) as second-line treatment during the same period at our center. The last follow-up was completed on June 30, 2025. The study was approved by the Ethics Committee of IHCAMS and conducted in accordance with the Declaration of Helsinki.

### Procedures

CTX was administered at a target dose of 400 mg/week (typically 6–8 mg/kg). For patients with baseline cytopenia (ANC < 1.0 × 10^9^/L or PLT < 20 × 10^9^/L), a fractional dose of 200 mg twice weekly or a reduced starting dose was employed. The median number of CTX doses administered was 4 (range: 1-12). Treatment was suspended if Grade 4 hematologic toxicity occurred and was not attributable to the primary disease. CTX was given either as monotherapy or in combination with other agents, including ruxolitinib, mesenchymal stem cells, basiliximab, or anti-CD25 monoclonal antibodies, among others. Treatment was continued until an overall response (OR) was achieved, unacceptable hematologic toxicity occurred, or no clinical benefit was observed. Mesna was routinely administered to prevent hemorrhagic cystitis. In the BAT cohort, patients received best available therapy without CTX, consisting of agents such as ruxolitinib, mesenchymal stem cells, basiliximab, anti-CD25 antibodies, or their combinations in our institution, as determined by the treating physician (**Supplementary Table 7**).

### End points and outcome assessment

The primary endpoint of the study was the ORR at day 28, which included both complete response (CR) and partial response (PR) rates. Secondary endpoints included durable ORR at day 56, overall survival (OS, defined as the time from CTX treatment to death from any cause, end of follow-up, or withdraw from study, whichever came first), failure-free survival (FFS, defined as the time from randomization to relapse or progression of hematologic disease, non-relapse-related death, or the initiation of new systemic therapy for hepatic aGVHD, whichever came first), non-relapse mortality (NRM, defined as the time from CTX administration to death from any cause without recurrence of the underlying disease), relapse of hematologic malignancies, and safety profile. Steroid refractoriness was assessed primarily according to widely accepted international guidelines (21). Treatment response was evaluated according to the clinical subtype of hepatic aGVHD: for the classic variant, response was defined by changes in total bilirubin levels; for the hepatitic variant, response was primarily assessed by the resolution of serum aminotransferase. Due to the lack of universally recognized staging criteria for the hepatitic variant of GVHD, a simplified working classification system for this variant of hepatic GVHD was adopted, as previously described in the literature: CR was defined as a reduction in aminotransferase levels to ≤2 × upper normal limit, while PR was defined as a reduction in aminotransferase levels to >2 × upper normal limit but <50% of the initial levels (22).

### Statistical analysis

Data were analyzed using R version 4.2.3 and GraphPad Prism version 9.5.0. Continuous variables were compared using the t-test or Wilcoxon rank-sum test; categorical variables were assessed with the Chi-square or Fisher’s exact test. Propensity score matching (PSM) was performed using the ‘MatchIt’ package with a 1:1 nearest-neighbor method and a caliper of 0.2, based on age, donor-recipient sex matching, donor type, conditioning regimen, GVHD prophylaxis and the severity of hepatic aGVHD. OS was estimated by the Kaplan-Meier method and compared using the log-rank test. Cox proportional hazards models were used for univariate and multivariate analyses of OS and FFS. Relapse and NRM were analyzed using competing risks models, with group comparisons by Fine-Gray’s test (23). Variables with *P* < 0.1 in univariate analysis were included in multivariate models. A two-sided *P* < 0.05 was considered statistically significant.

## Results

### Baseline characteristics

Fifty patients (median age [range], 34 [10-58] years; 18 [36.0%] women) who received CTX treatment were enrolled in this study, with the basic demographic characteristics listed in **Table 1**. Of these, 46 patients (92.0%) had hematologic malignancies and underwent myeloablative conditioning (MAC), while 4 patients (8.0%) with aplastic anemia received reduced-intensity conditioning (RIC). All patients underwent peripheral blood stem cell (PBSC) transplantation, and six patients (12.0%) received cord blood at +4 days post-HSCT due to poor graft quality. GVHD prophylaxis consisted of cyclosporine A or tacrolimus and short-term methotrexate for all patients, notably, PTCy was not used as GVHD prophylaxis regimen in this cohort. Recipients of HLA haploidentical donor (HID, [74.0%]) transplants received mycophenolate mofetil, consistent with prior experience, in contrast to recipients of HLA-matched sibling donor HSCT (MSD, 26.0%). Among the patients, 42 (84.0%) were diagnosed with the classic variant of hepatic aGVHD, and 30 of these (71.4%) exhibited stage 3–4 severity, while 8 (16.0%) had the hepatitic variant of aGVHD.

**Table 1:** Baseline and transplant characteristics of patients treated with cyclophosphamide.

Variables	Total (n = 50)
Age, M (Q_1_, Q_3_)	34.00 (24.25, 53.00)
Sex, n(%)	
Female	18 (36.00)
Male	32 (64.00)
Underling disease, n(%)	
Hematologic malignancies	46 (92.00)
Nonmalignant hematologic disease	4 (8.00)
Graft type, n(%)	
PB	44 (88.00)
PB+UB	6 (12.00)
Conditioning regimen, n(%)	
MAC	46 (92.00)
RIC	4 (8.00)
GVHD prophylaxis regimen, n(%)	
CsA based	31 (62.00)
FK506 based	19 (38.00)
Use of ATG, n(%)	
Yes	46 (92.00)
No	4 (8.00)
Donor age, M (Q_1_, Q_3_)	39.00 (31.00, 50.00)
Doner type, n(%)	
Haploidentical donor	37 (74.00)
Matched sibling donor	13 (26.00)
Donor-recipient sex matched, n(%)	
FF	6 (12.00)
FM	10 (20.00)
MF	13 (26.00)
MM	21 (42.00)
Overall severity of aGVHD*, n(%)	
Grade II	4 (8.00)
Grade III	14 (28.00)
Grade IV	30 (60.00)
Liver aGVHD after DLI, n(%)	
Yes	15 (30.00)
No	35 (70.00)
Type of liver aGVHD, n(%)	
Classic variant of aGVHD	42 (84.00)
Hepatitic variant of aGVHD	8 (16.00)
Severity of liver aGVHD before CTX treatment *, n(%)	
Stage 1	4 (8.00)
Stage 2	8 (16.00)
Stage 3	17 (34.00)
Stage 4	13 (26.00)
Organ involvement, n(%)	
Gastrointestinal tract	37 (74.00)
Skin	30 (60.00)

M, Median; Q_1_, 1st Quartile; Q_3_, 3st Quartile; PB, peripheral blood; UB, umbilical cord blood; MAC, myeloablative conditioning; RIC, reduced intensity conditioning; CsA, cyclosporine A; MTX, methotrexate; MMF, mycophenolate mofetil; FK506, tacrolimus; ATG, anti-thymocyte globulin; FF, female to female; FM, female to male; DLI, donor lymphocyte infusion; aGVHD, acute graft-versus-host disease.

*Hepatitic variant of liver GVHD is not involved in the evaluation.

CTX was administered as second-line therapy in 34.0% of patients and as third-line or later therapy in 66.0%. Due to the significant heterogeneity of the severity of hepatic aGVHD, PSM was performed in patients with the classic variant of hepatic aGVHD, yielding 35 matched pairs. Post-matching, baseline characteristics were well balanced (**Table 2**). Compared to the BAT group, CTX was initiated later (median: 8 vs. 2 days from hepatic aGVHD diagnosis; P < 0.001) and was more often used as later-line therapy (median line: 3 vs. 2; P < 0.001) after SR-hepatic aGVHD diagnosed.

**Table 2:** Patient and aGVHD characteristics in CTX and BAT groups: overall and propensity score-matched populations.

Variable	Before PSM					After PSM				
	Total (n = 124)	BAT (n = 82)	CTX (n = 42)	*P*	SMD	Total (n = 70)	BAT (n = 35)	CTX (n = 35)	*P*	SMD
Age, n (%)				0.251					0.550	
<55yrs	104 (83.87)	71 (86.59)	33 (78.57)		-0.195	56 (80)	29 (82.86)	27 (77.14)		-0.136
≥55yrs	20 (16.13)	11 (13.41)	9 (21.43)		0.195	14 (20)	6 (17.14)	8 (22.86)		0.136
Donor-recipient sex, n (%)				0.524					0.811	
uniformity	60 (48.39)	38 (46.34)	22 (52.38)		0.121	35 (50)	18 (51.43)	17 (48.57)		-0.057
disparity	64 (51.61)	44 (53.66)	20 (47.62)		-0.121	35 (50)	17 (48.57)	18 (51.43)		0.057
Donor type, n (%)				0.150					1.000	
MSD	40 (32.26)	30 (36.59)	10 (23.81)		-0.300	16 (22.86)	8 (22.86)	8 (22.86)		0.000
HID	84 (67.74)	52 (63.41)	32 (76.19)		0.300	54 (77.14)	27 (77.14)	27 (77.14)		0.000
Conditioning regimen, n (%)				1.000					1.000	
RIC	10 (8.06)	7 (8.54)	3 (7.14)		-0.054	6 (8.57)	3 (8.57)	3 (8.57)		0.000
MAC	114 (91.94)	75 (91.46)	39 (92.86)		0.054	64 (91.43)	32 (91.43)	32 (91.43)		0.000
Prophylaxis, n (%)				0.127					1.000	
FK506 based	65 (52.42)	47 (57.32)	18 (42.86)		-0.292	34 (48.57)	17 (48.57)	17 (48.57)		0.000
CSA based	59 (47.58)	35 (42.68)	24 (57.14)		0.292	36 (51.43)	18 (51.43)	18 (51.43)		0.000
Liver Severity, n (%)				0.006					1.000	
Stage 1	16 (12.9)	12 (14.63)	4 (9.52)		-0.174	7 (10)	3 (8.57)	4 (11.43)		0.090
Stage 2	45 (36.29)	37 (45.12)	8 (19.05)		-0.664	17 (24.29)	9 (25.71)	8 (22.86)		-0.068
Stage 3	40 (32.26)	23 (28.05)	17 (40.48)		0.253	28 (40)	14 (40.00)	14 (40.00)		0.000
Stage 4	23 (18.55)	10 (12.20)	13 (30.95)		0.406	18 (25.71)	9 (25.71)	9 (25.71)		0.000

PSM, propensity score matching; BAT, best available treatment; CTX, cyclophosphamide; SMD, standardized mean difference; HID, haploidentical donor; MSD, matched sibling donor; MAC, myeloablative conditioning; RIC, reduced intensity conditioning; CsA, cyclosporine A; FK506, tacrolimus.

### Response and long-term clinical outcomes

The median duration of CTX therapy was 21.5 days (range, 2–97). Following a median cumulative dose of 1,000 mg (range, 200–2,600 mg), 35 of 50 patients (70.0%) achieved OR at day 28, including 20 (40.0%) with CR and 15 (30.0%) with PR. The median time to response was 15 days (range, 2–63). Patients with the hepatitic variant of hepatic aGVHD were more likely to achieve CR than those with the classic variant (75.0% vs. 33.3%; *P* = 0.042). The day 56 durable ORR in the CTX group was 66.0%.

**Figure 2:**
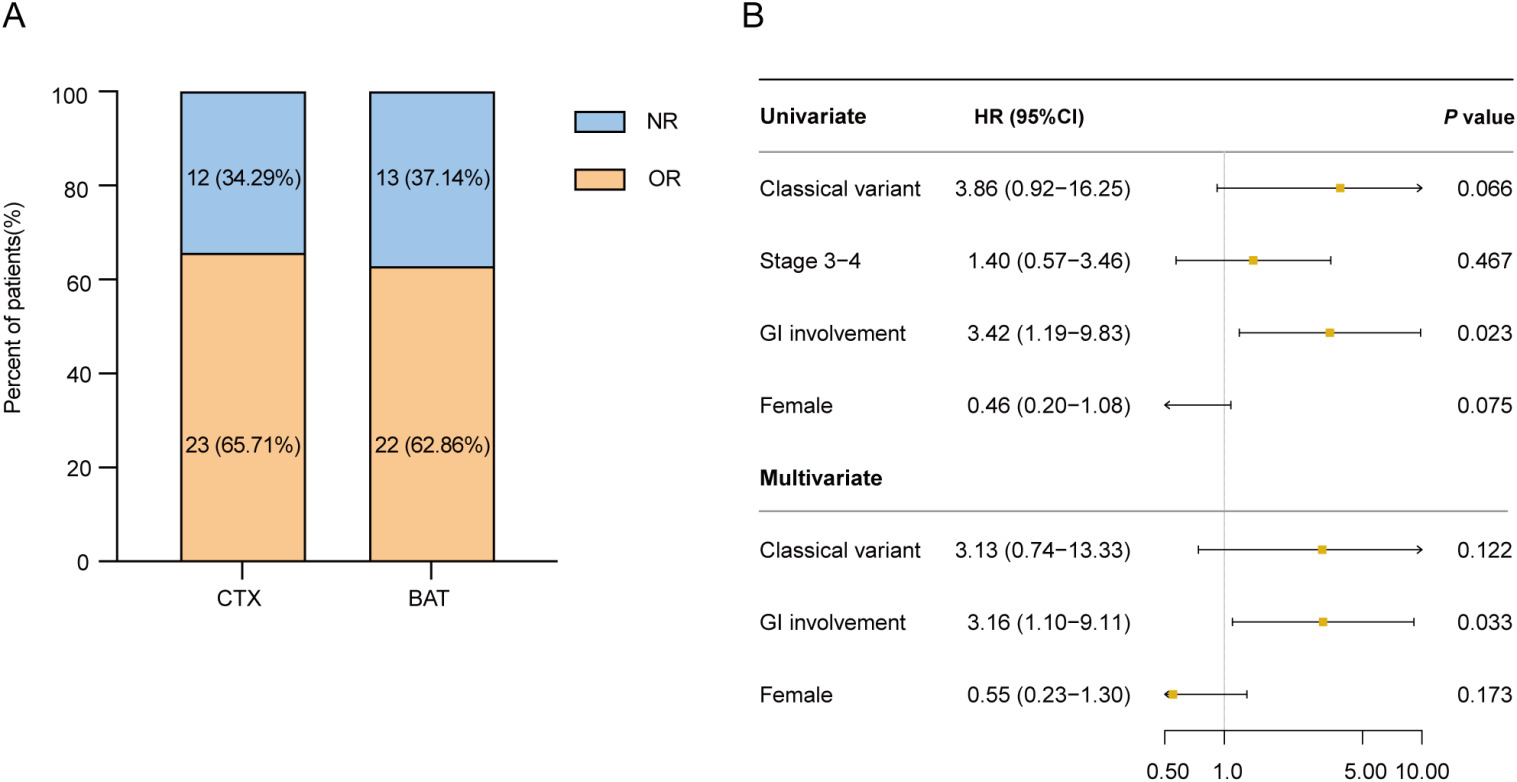
**(A)** Overall response (OR) and non-response (NR) rates of two groups at day28. **(B)** Univariate and multivariate analyses of factors associated with overall survival (OS) among patients treated with cyclophosphamide. OR, overall response; NR, non-response; CTX, cyclophosphamide; BAT, best available therapy; SR-aGVHD, steroid-refractory acute graft-versus-host disease; GI, gastrointestinal; HR, hazard ratio; CI, confidence interval.

In the matched cohort, there were no significant differences in day 28 ORR between the CTX and BAT groups (65.7% vs. 62.9%; *P* = 0.803; **Figure 2A**), nor in day 56 durable ORR (60.0% vs. 60.0%; *P* = 1.000). Given the higher proportion of patients receiving CTX as third-line or later therapy, we performed a subgroup analysis restricted to patients treated in the third-line setting. Baseline characteristics remained balanced (**Supplementary Table 1**), although the CTX group demonstrated a marginally higher response rate than the BAT group at day 28, the difference was not statistically significant (68.0% vs. 58.8%; *P* = 0.542).

**Figure 3:**
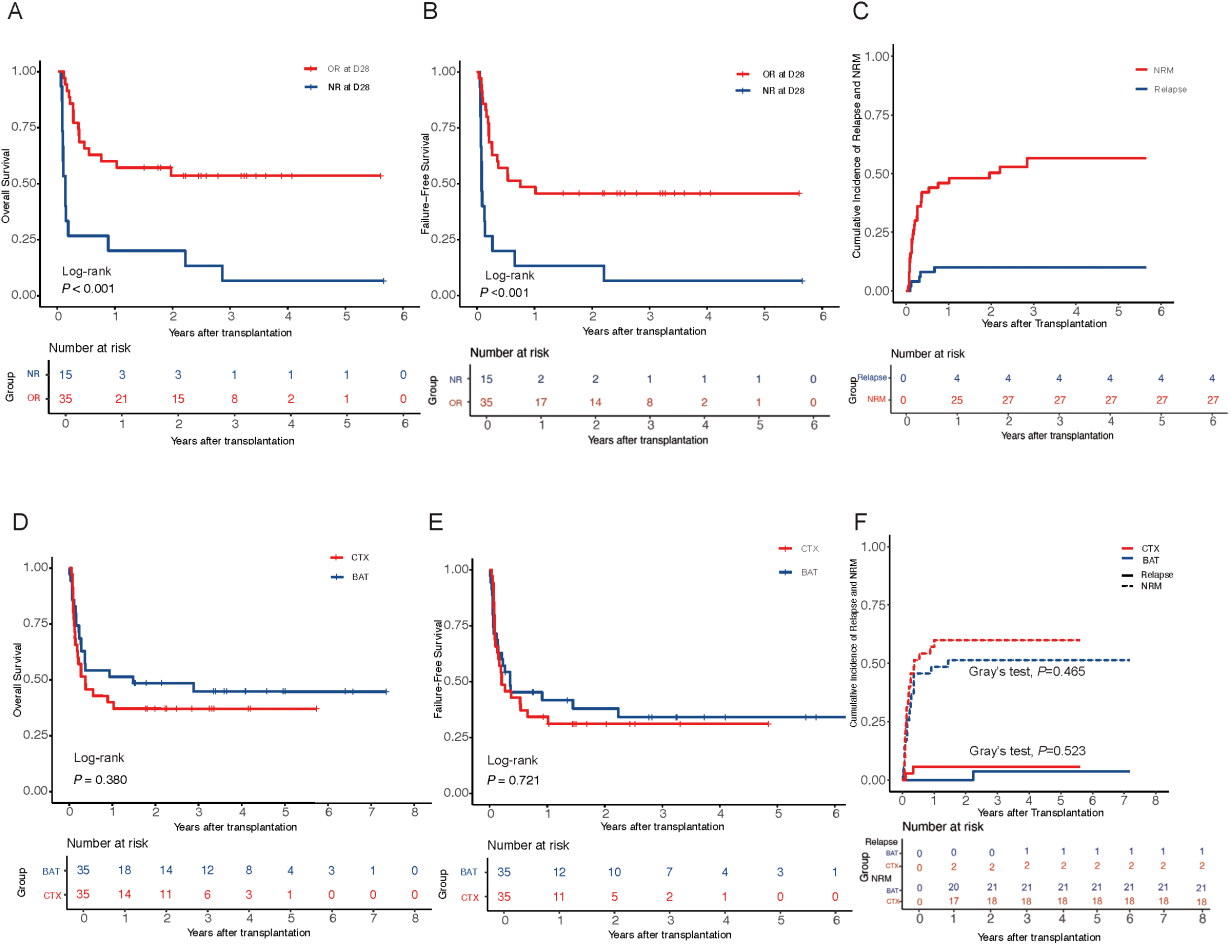
Survival outcomes and relapse/NRM incidences in hepatic SR-aGVHD patients. **(A, B)** Patients with overall response (OR) at day 28 showed significantly improved overall survival (OS) and failure-free survival (FFS) compared to non-responders (NR). **(C)** Cumulative incidence curves of relapse and non-relapse mortality (NRM) of patients treated with cyclophosphamide. **(D–F)** OS, FFS, cumulative incidence curves of relapse, and NRM between the CTX and BAT groups. OR, overall response; NR, non-response; OS, overall survival; FFS, failure-free survival; NRM, non-relapse mortality; CTX, cyclophosphamide; BAT, best available therapy; SR-aGVHD, steroid-refractory acute graft-versus-host disease.

The median follow-up duration was 2.7 years (range, 0.05–5.65 years). The estimated 3-year OS rate among all patients treated with CTX was 36.9% (95% CI, 24.8%–54.9%). Patients who achieved OR after CTX treatment had significantly better OS and FFS than non-responders. The 3-year cumulative incidence of relapse was 4.3% (95% CI, 1.6%–8.4%), while the 3-year NRM was 56.5% (95% CI, 41.4%–71.6%) (**Figures 3A–C**). Among 50 patients, 30 (30 of 50, 60.0%) patients eventually died. The primary cause of death was infection (11 out of 50, 36.6%), followed by aGVHD (7 out of 50, 23.3%) (**Supplementary Table 6**). In the matched cohort, no significant differences were observed between the CTX and BAT groups in 3-year OS (37.1% vs. 44.8%, P = 0.516), FFS (31.3% vs. 34.2%, P = 0.796), relapse (5.71% vs. 3.73%, P = 0.522), and NRM (60.0% vs. 51.4%, P = 0.465) (**Figures 3D–F**).

### Prognostic factors

To explore prognostic factors among patients treated with CTX, univariate and multivariate analyses were conducted. The effects of clinical and biological variables on OS are shown in **Figure 2B**. In multivariate analysis, gastrointestinal (GI) tract involvement by aGVHD was significantly associated with inferior OS (HR 3.16; 95% CI, 1.10–9.11; P = 0.033). A similar trend was observed for FFS (**Supplementary Table 2**). Female sex appeared to be a favorable factor, although the difference was not statistically significant. We also performed uni- and multivariate analyses in the post-PSM cohort, including patients from both the CTX and BAT groups. Results were consistent, with stage 3–4 hepatic aGVHD emerging as a potential adverse prognostic factor for both OS and FFS (**Supplementary Tables 3**, **4**).

### Safety profile

Hematologic toxicity was the most common adverse event within 28 days following CTX administration, with anemia and neutropenia occurring in 35 (70.0%) and 31 (62.0%) of 50 patients, respectively. Grade 3–4 anemia was reported in 29 patients (58.0%), grade 3–4 thrombocytopenia in 22 (44.0%), and grade 3–4 neutropenia in 18 (36.0%).

Infectious complications were also frequent, affecting 38 patients (76.0%, **Supplementary Table 5**). The most common infection was cytomegalovirus (CMV), identified in 25 patients (50.0%), followed by pulmonary infections in 14 patients (28.0%). Among pulmonary infections, 6 (12.0%) were fungal, 5 (10.0%) bacterial, 1 (2.0%) viral, and 2 (4.0%) mixed etiology (**Supplementary Table 5**). Subgroup analysis revealed that the initial dose (400 mg vs. 200 mg) did not significantly impact the incidence of Grade 3–4 hematologic toxicities (P = 0.791) or CMV reactivation (P = 0.510).

When compared with the BAT group, CTX did not significantly increase the incidence of adverse events within 28 days. There were no statistically significant differences in the rates of neutropenia (71.4% vs. 62.9%, P = 0.445), anemia (68.6% vs. 60.0%, P = 0.454), or cytomegalovirus infection (51.4% vs. 45.7%, P = 0.632) between the CTX and BAT groups.

## Discussion

Post-transplant hepatic dysfunction occurs in approximately 50% to 80% of patients undergoing allo-HSCT, with hepatic aGVHD representing a major contributor to this complication and a known determinant of NRM and inferior OS (24, 25). Standard first-line treatment with high-dose corticosteroids, often combined with a calcineurin inhibitor, yields suboptimal responses in hepatic aGVHD, particularly in advanced stages (26). Multiple second-line agents such as ruxolitinib, anti-cytokine antibodies, or cellular therapies have been used, however, the liver is often the least responsive organ among those affected by aGVHD, for example, ruxolitinib yields a liver response rate of only 26.7% at day 28 (12, 27, 28). This disparity is primarily due to differences in pathogenesis across organs, with Fas ligands potentially mediating hepatic aGVHD, while skin and gut aGVHD may involve perforin and granzyme pathways (29, 30). In this context, our study explored the clinical utility of CTX, a widely available and cost-effective immunosuppressive agent with known efficacy in GVHD prophylaxis (31), as salvage therapy in SR-hepatic aGVHD.

Among 50 patients treated with CTX, the ORR at day 28 reached 70.0%, with a CR rate of 40.0%. These outcomes are notable, particularly given that 66.0% of patients received CTX as third-line or later therapy, and they compare favorably to previously reported results with ruxolitinib. Due to the heterogeneity of patient populations and variations in treatment regimens, direct comparisons between therapies were not feasible. Therefore, we compared the effects of other second-line therapies administered during the same period for treating hepatic aGVHD. In our PSM analysis with a control group receiving BAT, CTX and BAT demonstrated similar efficacy across multiple endpoints, including ORR at day 28, durable response at day 56, OS, FFS, and NRM. However, the CTX cohort had a median treatment delay of 6 days and included a higher proportion of patients receiving later-line therapy, suggesting that CTX retained clinical activity even in advanced and refractory settings. Importantly, these findings reposition CTX not as a superior agent but as a feasible, safe, and accessible option for heavily pretreated patients with limited alternatives.

We further observed a significantly higher CR rate in patients with the hepatitic variant of hepatic aGVHD compared to the classic variant (75.0% vs. 33.3%, P = 0.042). This trend persisted at day 56, aligning with the findings of previous studies (18). The hepatitic variant, characterized by marked transaminase elevation (>10 × ULN) and limited bilirubin or alkaline phosphatase elevation, is underrepresented in standard grading systems and often clinically underestimated. Pathologically, the hepatitic variant displays prominent lobular necroinflammation and acidophilic body formation, which may reflect increased susceptibility to the cytotoxic and immunomodulatory effects of CTX, as the intense T-cell proliferation characteristic of this variant provides more cellular targets for CTX-mediated apoptosis. Its frequent association with donor lymphocyte infusion (DLI) or immunosuppressant tapering may also suggest heightened alloimmune reactivity that is responsive to CTX-mediated suppression (29).

Previous studies have demonstrated that concurrent hepatic and GI involvement in aGVHD is associated with inferior survival outcomes (32). Consistent with these findings, our study identified GI involvement in 74% of patients, which emerged as a significant independent risk factor for poor prognosis, in multivariate analysis, GI aGVHD was associated with worse OS (HR 3.16, *P* = 0.033). Pathophysiologically, GI tract damage compromises mucosal integrity, facilitating microbial translocation, systemic inflammation, and cytokine dysregulation—all of which may exacerbate hepatic injury and attenuate response to immunosuppressive therapy (33). Additionally, impaired nutrient absorption and altered drug metabolism may further undermine treatment tolerance and efficacy. These results underscore the need for intensified or combinatorial approaches in patients with overlapping GI and hepatic aGVHD. The 3-year NRM was 56.5%, comparable to that in the BAT group. This elevated rate likely reflects advanced disease status, delayed intervention, and frequent multi-organ involvement.

CTX was generally well tolerated. Myelosuppression was the most common toxicity, with 70.0% of patients experiencing anemia and 62.0% developing neutropenia, though these rates were not significantly different from those observed in the BAT group. CMV reactivation occurred in 50.0% of patients but was manageable. In our safety analysis, no significant dose-response relationship was observed regarding severe adverse events. The rates of Grade 3–4 hematologic toxicities and CMV reactivation remained comparable between patients receiving 400 mg and those receiving 200 mg. These findings indicate that while CTX contributes to the overall immunosuppressive burden, its toxicity profile appears stable within the current dosing range. Previous reports have also described the use of CTX in combination treatments for cGVHD with modest myelotoxicity (34). Although the precise mechanisms remain to be fully elucidated, existing evidence suggests that CTX may exert therapeutic effects in SR-hepatic aGVHD by selectively depleting highly activated alloreactive T cells while relatively sparing regulatory T cells. This immunomodulatory effect may help restore immune balance within the hepatic microenvironment, which is characterized by intense local T-cell–mediated inflammation. By interrupting inflammatory amplification and attenuating immune-mediated bile duct injury, CTX contributes to the improvement of cholestatic liver dysfunction observed in hepatic aGVHD. The relative preservation of regulatory T cells further facilitates immune reconstitution and promotes intrahepatic immune tolerance (16, 35–37). These properties distinguish CTX from other agents with broader immunosuppressive effects.

Our study has several limitations. As a retrospective, single-center analysis, it is subject to inherent selection bias and a limited sample size—particularly within hepatic aGVHD phenotypic subgroups—which may restrict statistical power for detecting moderate treatment effects and conducting robust subgroup or interaction analyses. Furthermore, our analysis focused exclusively on hepatic aGVHD, consistent with prior case-based reports that informed the use of CTX in this setting, whether CTX exerts clinically meaningful effects on extra-hepatic SR-aGVHD deserves further investigation. Accordingly, prospective multicenter trials with larger cohorts, ideally stratified by hepatic variant and concurrent GI involvement, are warranted to better define the therapeutic role of CTX, optimize dosing strategies, and evaluate its potential in combination regimens.

## Conclusions

In conclusion, CTX is a feasible, well-tolerated, and accessible salvage option for SR-hepatic aGVHD, warranting consideration in future treatment strategies.

## Data Availability

The raw data supporting the conclusions of this article will be made available by the authors, without undue reservation.
