# Supplementary material for: Evaluation of cyclophosphamide for steroid-refractory hepatic acute graft-vs-host disease after allogeneic hematopoietic stem cell transplantation
Source: Front Immunol. 2026 Mar 2;17:1678723. doi: 10.3389/fimmu.2026.1678723 (PMC12989407; doi:10.3389/fimmu.2026.1678723)
Supplement: Supplementary file 1 [file Table1.docx]

**Table S1. Baseline Characteristics of Advanced-Line (≥3) Therapy Patients in CTX and BAT Cohorts.**

| Variables | Total  (n = 42) | BAT  (n = 17) | CTX  (n = 25) | P value |
| --- | --- | --- | --- | --- |
|  |  |  |  |  |
| Age, n (%) |  |  |  | 0.237 |
| ＜55yrs | 34 (82.93) | 16 (94.12) | 18 (75.00) |  |
| ≥55yrs | 7 (17.07) | 1 (5.88) | 6 (25.00) |  |
| Sex, n(%) |  |  |  | 0.650 |
| Male | 24 (57.14) | 9 (52.94) | 15 (60.00) |  |
| Female | 18 (42.86) | 8 (47.06) | 10 (40.00) |  |
| Conditioning regimen, n(%) |  |  |  | 1.000 |
| MAC | 4 (9.52) | 2 (11.76) | 2 (8.00) |  |
| RIC | 38 (90.48) | 15 (88.24) | 23 (92.00) |  |
| Doner type, n(%) |  |  |  | 0.834 |
| Haploidentical donor | 8 (19.05) | 4 (23.53) | 4 (16.00) |  |
| Matched sibling donor | 34 (80.95) | 13 (76.47) | 21 (84.00) |  |
| Donor-recipient sex matched, n(%) |  |  |  | 0.856 |
| match | 18 (42.86) | 7 (41.18) | 11 (44.00) |  |
| mismatch | 24 (57.14) | 10 (58.82) | 14 (56.00) |  |

*Abbreviations*: BAT: best available treatment; CTX: cyclophosphamide； MAC: myeloablative conditioning; RIC: reduced intensity conditioning.

**Table S2. Univariate and multivariate analyses of FFS for 50 patients treated with CTX.**

| Variables | Univariate | | Multivariate | |
| --- | --- | --- | --- | --- |
|  | P value | HR (95%CI) | P value | HR (95%CI) |
| Liver type |  |  |  |  |
| Hepatitis variant |  | 1.00 (Reference) |  |  |
| Classical variant | 0.106 | 2.67 (0.81 ~ 8.75) |  |  |
| Liver severity |  |  |  |  |
| Stage 1-2 |  | 1.00 (Reference) |  |  |
| Stage 3-4 | 0.859 | 1.08 (0.48 ~ 2.42) |  |  |
| GI involvement |  |  |  |  |
| No |  | 1.00 (Reference) |  | 1.00 (Reference) |
| Yes | 0.070 | 2.27 (0.93 ~ 5.53) | 0.070 | 2.27 (0.93 ~ 5.53) |
| Sex |  |  |  |  |
| Male |  | 1.00 (Reference) |  |  |
| Female | 0.206 | 0.61 (0.28 ~ 1.31) |  |  |

*Abbreviations*: FFS: failure‐free survival; GI: gastrointestinal tract; HR: hazard ratio.

**Table S3. Univariate and multivariate analyses of OS for the CTX and BAT groups.**

| Variables | Univariate | | Multivariate | |
| --- | --- | --- | --- | --- |
|  | P value | HR (95%CI) | P value | HR (95%CI) |
| Sex |  |  |  |  |
| Male |  | 1.00 (Reference) |  |  |
| Female | 0.940 | 0.98 (0.52 ~ 1.83) |  |  |
| Liver severity |  |  |  |  |
| Stage 1-2 |  | 1.00 (Reference) |  | 1.00 (Reference) |
| Stage 3-4 | 0.050 | 2.00 (1.00 ~ 3.99) | 0.0872 | 1.84 (0.915 ~ 3.71) |
| Group |  |  |  |  |
| BAT |  | 1.00 (Reference) |  | 1.00 (Reference) |
| CTX | 0.383 | 1.32 (0.71 ~ 2.43) | 0.445 | 1.27 (0.684 ~ 2.37) |
| GI involvement |  |  |  |  |
| No |  | 1.00 (Reference) |  | 1.00 (Reference) |
| Yes | 0.012 | 4.52 (1.39 ~ 14.68) | 0.0135 | 4.43 (1.36 ~ 14.4) |

*Abbreviations*: OS: overall survival; BAT: best available treatment; CTX: cyclophosphamide; GI: gastrointestinal tract; HR: hazard ratio.

**Table S4. Univariate and multivariate analyses of FFS for the CTX and BAT groups.**

| Variables | Univariate | | Multivariate | |
| --- | --- | --- | --- | --- |
|  | P value | HR (95%CI) | P value | HR (95%CI) |
| Sex |  |  |  |  |
| Male |  | 1.00 (Reference) |  |  |
| Female | 0.768 | 1.09 (0.61 ~ 1.97) |  |  |
| Liver severity |  |  |  |  |
| Stage 1-2 |  | 1.00 (Reference) |  | 1.00 (Reference) |
| Stage 3-4 | 0.074 | 1.80 (0.94 ~ 3.42) | 0.121 | 1.68 (0.872 ~ 3.24) |
| Group |  |  |  |  |
| BAT |  | 1.00 (Reference) |  | 1.00 (Reference) |
| CTX | 0.718 | 1.11 (0.62 ~ 1.99) | 0.857 | 1.06 (0.585 ~ 1.90) |
| GI involvement |  |  |  |  |
| No |  | 1.00 (Reference) |  | 1.00 (Reference) |
| Yes | 0.028 | 2.84 (1.12 ~ 7.22) | 0.0363 | 2.72 (1.07 ~ 6.94) |

*Abbreviations*: FFS: failure‐free survival; BAT: best available treatment; CTX: cyclophosphamide; GI: gastrointestinal tract; HR: hazard ratio.

**Table S5. Summary of infections after CTX treatment.**

| Types of infection | n(%) |
| --- | --- |
|  |  |
| Lung infection  Intracranial infection  Cutaneous tissue infection  Cytomegalovirus infection  Epstein-Barr virus infection  Other infection  Any infection (≥ 1 type) | 14 (28.00)  2 (4.00)  4 (8.00)  25 (50.00)  6 (12.00)  3 (6.00)  38(76.00) |
|  | |

**Table S6. Summary of death reasons after CTX treatment.**

| Cause of death | n(%) |
| --- | --- |
|  |  |
| Relapse  Non-relapse mortality  Infection  aGVHD  Hemorrhage  Multiple organ dysfunction | 4 (13.33)  26 (86.67)  11 (36.67)  7 (23.33)  5 (16.67)  3 (10.00) |
| *Abbreviations*: aGVHD: acute graft-versus-host disease | |

**Table S7. Individual treatment timelines and clinical course for patients receiving CTX or BAT as salvage therapy.**

| **ID** | **Group** | **GVHD prophylaxis** | **Grade of aGVHD** | **Liver aGVHD Stage** | **Other organs involvement** | **Liver aGVHD after HSCT（d）** | **Hepatic aGVHD therapies** | **From diagnose to treatment** | **Overall response** | **Response at D28** | **Response at D56** | **Survival status** |
| --- | --- | --- | --- | --- | --- | --- | --- | --- | --- | --- | --- | --- |
| 1 | BAT | CsA+MMF | 3 | 3 | GI+Skin | 24 | MSC+BSX+IFX+RUX+ETN | 0 | CR | CR | CR | survival |
| 2 | BAT | FK506+MMF | 4 | 4 | GI+Skin | 224 | BSX | 15 | NR | NR | NR | death |
| 3 | BAT | CsA+MTX | 4 | 4 | GI+Skin | 42 | ETN+BSX+MSC+RUX | 5 | NR | NR | NR | death |
| 4 | BAT | CsA+MZR | 4 | 2 | GI+Skin | 32 | BSX+IFX+MSC+RUX | 0 | CR | NR | CR | survival |
| 5 | BAT | CsA+MTX+MMF | 3 | 2 | GI+Skin | 43 | BSX+MSC+RUX | 0 | CR | CR | CR | death |
| 6 | BAT | FK506+MMF | 4 | 3 | GI+Skin | 37 | ETN+MSC | 4 | NR | NR | NR | death |
| 7 | BAT | CsA+MMF | 3 | 1 | GI+Skin | 20 | MSC+ETN | 2 | CR | CR | CR | survival |
| 8 | BAT | CsA+MMF+MTX+MSC | 3 | 2 | GI | 56 | RUX+MSC | 0 | CR | CR | NR | death |
| 9 | BAT | FK506+MMF | 3 | 1 | Skin | 21 | BSX | 0 | CR | CR | CR | death |
| 10 | BAT | FK506+MMF | 4 | 4 | GI | 55 | BSX+MSC+RUX | 6 | CR | PR | PR | survival |
| 11 | BAT | CsA+MMF | 3 | 2 | GI+Skin | 95 | BSX+RUX | 6 | CR | CR | CR | death |
| 12 | BAT | CsA | 3 | 3 | None | 100 | RUX+ETN | 4 | CR | CR | CR | survival |
| 13 | BAT | CsA | 3 | 1 | GI | 48 | ETN+BSX+MSC | 3 | CR | CR | CR | survival |
| 14 | BAT | CsA | 3 | 2 | GI | 84 | BSX+RUX+Anti-CD25 | 4 | CR | CR | CR | survival |
| 15 | BAT | CsA+MMF+MTX | 3 | 3 | GI | 33 | RUX+TNF-α+IFX | 0 | NR | NR | NR | death |
| 16 | BAT | CsA+MMF | 3 | 2 | GI | 25 | RUX | 1 | NR | NR | NR | death |
| 17 | BAT | FK506+MTX+MMF | 4 | 2 | GI+Skin | 65 | Anti-CD25+MSC | 2 | CR | CR | CR | survival |
| 18 | BAT | FK506+MTX+MMF | 4 | 4 | GI | 102 | Anti-CD25 | 7 | NR | NR | NR | death |
| 19 | BAT | FK506+MTX+MMF | 3 | 2 | None | 114 | RUX | 1 | CR | CR | CR | survival |
| 20 | BAT | CsA | 4 | 3 | GI+Skin | 49 | MSC+Anti-CD25+IFX+RUX | 1 | CR | CR | PR | survival |
| 21 | BAT | FK506+MTX | 4 | 3 | GI+Skin | 54 | Anti-CD25+MSC+RUX | 1 | CR | CR | CR | survival |
| 22 | BAT | FK506++MMF+MTX | 4 | 4 | GI+Skin | 32 | ETN+MSC+RUX+BSX+RUX | 0 | NR | NR | NR | death |
| 23 | BAT | CsA+MMF+MTX | 4 | 4 | GI+Skin | 58 | MSC+BSX+IFX | 0 | NR | NR | NR | death |
| 24 | BAT | FK506+MMF | 4 | 4 | Skin+Mouth | 48 | ADA+Anti-CD25 | 9 | PR | PR | PR | survival |
| 25 | BAT | CsA | 4 | 4 | GI+Skin | 28 | BSX+ETN+MSC+ATG | 3 | NR | NR | NR | death |
| 26 | BAT | FK506+MMF | 4 | 4 | GI | 22 | RUX | 5 | NR | NR | NR | death |
| 27 | BAT | CsA | 3 | 2 | None | 41 | RUX | 4 | CR | CR | CR | survival |
| 28 | BAT | CsA | 4 | 3 | GI+Skin | 55 | BSX+IFX+MSC | 2 | PR | PR | PR | death |
| 29 | BAT | CsA | 3 | 3 | GI+Skin | 32 | RUX+BSX | 6 | CR | CR | CR | death |
| 30 | BAT | FK506+MMF | 4 | 3 | GI | 45 | RUX+BSX | 1 | CR | CR | CR | death |
| 31 | BAT | FK506 | 3 | 3 | Skin | 97 | MSC | 22 | CR | CR | CR | survival |
| 32 | BAT | FK506+MMF | 3 | 3 | GI+Skin | 46 | Anti-CD25 | 12 | NR | NR | NR | death |
| 33 | BAT | FK506+MMF | 3 | 2 | GI+Skin | 57 | RUX+BSX | 5 | CR | CR | CR | survival |
| 34 | BAT | FK506+MMF | 4 | 4 | GI | 15 | BSX+MSC+ETN | 1 | NR | NR | NR | death |
| 35 | BAT | FK506+MTX | 3 | 3 | GI | 100 | RUX+BSX | 0 | CR | PR | CR | survival |
| 36 | CTX | FK506+MMF+MTX | 4 | 3 | GI+Skin | 30 | BSX+ADA+MSC+CTX | 7 | NR | NR | NR | death |
| 37 | CTX | CsA+MTX+MMF | 4 | 2 | GI | 42 | MMF+RUX+Anti-CD25+MSC+ETN+CTX | 8 | PR | PR | NR | death |
| 38 | CTX | CsA+MMF+MTX | 4 | 3 | GI | 35 | RUX+Anti-CD25+MSC+MMF+CTX | 31 | CR | CR | PR | death |
| 39 | CTX | FK506+MMF＋MTX | 3 | 2 | GI | 74 | RUX+BSX+CTX | 14 | CR | CR | NR | death |
| 40 | CTX | FK506+MMF+MTX | 4 | 4 | Skin | 95 | Anti-CD25+ETN+RTX+CTX | 18 | NR | NR | NR | death |
| 41 | CTX | CsA+MMF+MTX | 4 | 3 | GI | 46 | BSX+ETN+RUX+MMF+CTX | 30 | CR | CR | CR | death |
| 42 | CTX | FK506+MMF+MTX | 4 | 3 | GI | 55 | BSX+ETN+CTX | 58 | CR | CR | CR | death |
| 43 | CTX | CsA+MTX+MMF | 2 | 1 | GI | 46 | RUX+Anti-CD25+CTX | 3 | CR | CR | CR | survival |
| 44 | CTX | CsA+MMF+MTX | 3 | 2 | Skin | 53 | CTX | 6 | CR | CR | CR | survival |
| 45 | CTX | CsA+MTX | 3 | 3 | GI | 24 | BSX+CTX | 2 | CR | CR | CR | survival |
| 46 | CTX | CsA+MTX | 4 | 3 | GI+Skin | 77 | RUX+Anti-CD25+MSC+CTX | 30 | NR | NR | NR | death |
| 47 | CTX | CsA+MMF+MTX | 3 | 2 | Skin | 62 | RUX+Anti-CD25+CTX | 2 | CR | CR | CR | survival |
| 48 | CTX | FK506+MMF+MTX | 3 | 3 | Skin+Mouth | 40 | BSX+RUX+CTX | 10 | CR | PR | CR | survival |
| 49 | CTX | CsA+MTX+MMF+Ruxolitinib | 4 | 3 | GI+Skin | 32 | RUX+MSC+BSX+IFX+CTX | 3 | NR | NR | NR | death |
| 50 | CTX | CsA+MMF+MTX | 2 | 1 | GI | 52 | RUX+MSC+BSX+CTX | 3 | CR | CR | PR | survival |
| 51 | CTX | CsA+MTX | 2 | 1 | Skin | 44 | RUX+CTX | 19 | CR | CR | CR | survival |
| 52 | CTX | FK506+MMF+MTX | 3 | 3 | Skin | 44 | RUX+BSX+CTX | 7 | PR | PR | PR | survival |
| 53 | CTX | FK506+MMF+MTX | 4 | 4 | GI+Skin | 14 | Anti-CD25+MSC+CTX | 24 | NR | NR | NR | death |
| 54 | CTX | CsA+MTX | 4 | 4 | GI | 62 | ADA+MSC+CTX | 6 | NR | NR | NR | death |
| 55 | CTX | CsA+MMF+MTX | 3 | 2 | GI | 59 | Anti-CD25+RUX+CTX | 6 | CR | CR | CR | death |
| 56 | CTX | FK506+MMF+MTX | 4 | 4 | GI | 142 | BSX+MSC+CTX | 16 | NR | NR | NR | death |
| 57 | CTX | CsA+MTX+MMF | 4 | 2 | GI | 145 | RUX+CTX | 7 | NR | NR | NR | death |
| 58 | CTX | CsA+MMF+MTX | 3 | 2 | GI+Skin+Mouth | 89 | RUX+Anti-CD25+CTX | 65 | PR | PR | CR | survival |
| 59 | CTX | FK506+MMF+MTX | 4 | 3 | GI | 28 | RUX+BSX+MSC+MMF+CTX | 191 | NR | NR | NR | death |
| 60 | CTX | FK506++MMF+MTX | 3 | 3 | GI+Skin | 15 | Anti-CD25+CTX | 8 | PR | PR | PR | death |
| 61 | CTX | FK506+MMF+MTX | 3 | 3 | GI+Skin | 20 | RUX+Anti-CD25+MSC+CTX | 4 | CR | PR | CR | survival |
| 62 | CTX | CsA+MMF+MTX | 4 | 4 | GI+Skin+Mouth | 33 | Anti-CD25+RUX+ETN+CTX | 38 | CR | CR | CR | survival |
| 63 | CTX | CsA+MTX | 4 | 2 | GI+Skin+Eyes+Mouth | 44 | RUX+Anti-CD25+MSC+IFX+CTX | 3 | NR | NR | NR | death |
| 64 | CTX | FK506+MTX | 4 | 1 | GI | 25 | RUX+Anti-CD25+CTX | 0 | CR | CR | CR | death |
| 65 | CTX | FK506+MTX+MMF | 4 | 4 | GI+Skin | 31 | RUX+MSC+Anti-CD25+VDZ+CTX | 6 | PR | PR | PR | death |
| 66 | CTX | CsA+MMF+MTX | 3 | 3 | GI+Skin | 88 | RUX+Anti-CD25+CTX | 8 | CR | PR | CR | survival |
| 67 | CTX | FK506+MMF+MTX | 4 | 4 | GI+Skin | 16 | Anti-CD25+MSC+ETN+MMF+CTX | 35 | PR | PR | PR | death |
| 68 | CTX | FK506+MMF+MTX | 3 | 3 | None | 13 | RUX+CSA+Anti-CD25+CTX | 6 | CR | NR | PR | death |
| 69 | CTX | CsA+MMF+MTX | 4 | 4 | GI+Skin | 59 | BSX+ Anti-CD25+TNF-α+CTX | 39 | CR | PR | PR | survival |
| 70 | CTX | FK506+MTX+MMF | 4 | 4 | GI | 49 | MSC+ENT+CTX | 12 | PR | NR | PR | death |

*Abbreviations*: ADA: adalimumab; BSX: basiliximab; ETN: etanercept; IFX: infliximab; RUX: ruxolitinib; VDZ: vedolizumab.
